# HIV postnatal prophylaxis and infant feeding policies vary across Europe: results of a Penta survey

**DOI:** 10.1111/hiv.13723

**Published:** 2024-10-23

**Authors:** Georgina Fernandes, Elizabeth Chappell, Tessa Goetghebuer, Christian R. Kahlert, Santa Ansone, Stefania Bernardi, Guido Castelli Gattinara, Elena Chiappini, Catherine Dollfus, Pierre Frange, Bridget Freyne, Luisa Galli, Vania Giacomet, Galia Grisaru‐Soen, Christoph Königs, Hermione Lyall, Magdalena Marczynska, Mariana Mardarescu, Lars Naver, Tim Niehues, Antoni Noguera‐Julian, Kim Stol, Alla Volokha, Steven B. Welch, Claire Thorne, Alasdair Bamford

**Affiliations:** ^1^ Population, Policy and Practice Research and Teaching Department UCL Great Ormond Street Institute of Child Health London UK; ^2^ MRC Clinical Trials Unit at UCL London UK; ^3^ Hôpital Saint Pierre Université Libre de Bruxelles Brussels Belgium; ^4^ Infectious Diseases and Hospital Epidemiology Children's Hospital of Eastern Switzerland St. Gallen Switzerland; ^5^ Outpatient Department, Riga East University Hospital Latvian Centre of Infectious Diseases Riga Latvia; ^6^ Simplex Unit: Perinatal and Complex Infectious Disease – University Paediatric Clinical Area Bambino Gesù Children's Hospital Rome Italy; ^7^ Infectious Diseases Unit Meyer Children's University Hospital IRCCS Florence Italy; ^8^ Service d'Hémato‐Oncologie Pédiatrique APHP, Hopital Trousseau Paris France; ^9^ Laboratory of Clinical Microbiology Necker Enfants Malades Hospital, Groupe hospitalier APHP. Centre – Université Paris Cité Paris France; ^10^ Department of Paediatric Infectious Diseases Children's Health Ireland Dublin Ireland; ^11^ CEPHR, School of Medicine University College Dublin Dublin Ireland; ^12^ Department of Paediatrics, Paediatric Infectious Disease Unit, L Sacco Hospital University of Milan Milan Italy; ^13^ Pediatric Infectious Diseases Unit Dana Children's Hospital, Tel Aviv Sourasky Medical Center Tel Aviv Israel; ^14^ Department of Paediatrics and Adolescent Medicine Goethe University, University Hospital Frankfurt Frankfurt Germany; ^15^ Paediatric Infectious Diseases Imperial College Healthcare NHS Trust London UK; ^16^ Department of Children's Infectious Diseases Medical University of Warsaw Warsaw Poland; ^17^ Pediatric Department National Institute for Infectious Diseases ‘Prof. Dr. Matei Bals’ Bucharest Romania; ^18^ Department of Pediatrics, Karolinska University Hospital and Division of Pediatrics, Department of Clinical Science, Intervention and Technology (CLINTEC) Karolinska Institutet Stockholm Sweden; ^19^ Department of Pediatrics Helios Klinikum Krefeld Krefeld Germany; ^20^ Infectious Diseases and Systemic Inflammatory Response in Pediatrics, Pediatric Infectious Diseases Department, Institut de Recerca Sant Joan de Déu Hospital Sant Joan de Déu Barcelona Spain; ^21^ Centre for Biomedical Network Research on Epidemiology and Public Health (CIBERESP) Madrid Spain; ^22^ Departament de Cirurgia i Especialitats Medicoquirúrgiques, Facultat de Medicina i Ciències de la Salut Universitat de Barcelona Barcelona Spain; ^23^ Department of Pediatrics, Radboud University Nijmegen Medical Center Amalia Children's Hospital Nijmegen The Netherlands; ^24^ Department of Pediatrics, Pediatrics Infectious Diseases Immunology and Allergology Shupyk National Healthcare University of Ukraine Kyiv Ukraine; ^25^ Department of Paediatrics Heartlands Hospital University Hospitals Birmingham NHS Foundation Trust Birmingham UK; ^26^ Department of Paediatric Infectious Diseases Great Ormond Street Hospital for Children NHS Foundation Trust London UK; ^27^ Infection, Immunity and Inflammation Research and Teaching Department UCL Great Ormond Street Institute of Child Health London UK

**Keywords:** breastfeeding, HIV, paediatrics, policy, postnatal prophylaxis, vertical transmission

## Abstract

**Objectives:**

This survey was conducted to describe current European postnatal prophylaxis (PNP) and infant feeding policies with the aim of informing future harmonized guidelines.

**Methods:**

A total of 32 senior clinicians with relevant expertise, working in 20 countries within the European Region, were invited to complete a REDCap questionnaire between July and September 2023.

**Results:**

Twenty‐three of the 32 invited paediatricians responded, representing 16/20 countries. There were multiple respondents from the same country for Italy (*n* = 5), the UK (*n* = 2), Germany (*n* = 2) and France (*n* = 2). All countries use risk stratification to guide PNP regimen selection. Nine out of 16 countries reported three risk categories, six out of 16 reported two, and one country reported differences in categorization. Criteria used to stratify risk varied between and within countries. For the lowest risk category, the PNP regimen reported ranged from no PNP to up to four weeks of one drug; the drug of choice reported was zidovudine, apart from one country which reported nevirapine. For the highest risk category, the most common regimen was zidovudine/lamivudine/nevirapine (20/23 respondents); regimen duration varied from two to six weeks with variation in recommended dosing. Guidelines support breastfeeding for infants born to people living with HIV in eight out of 16 countries; in the other eight, guidelines do not support/specify.

**Conclusions:**

Guidelines and practice for PNP and infant feeding vary substantially across Europe and within some countries, reflecting the lack of robust evidence. Effort is needed to align policies and practice to reflect up‐to‐date knowledge to ensure the vertical transmission risk is minimized and unnecessary infant HIV testing and PNP avoided, while simultaneously supporting families to make informed decisions on infant feeding choice.

## INTRODUCTION

In Europe, rates of vertical transmission (VT) are now very low, with estimates ranging from 0.2% to 1.1% in recent reports [1, 2, 3, 4, 5, 6]. This reflects a combination of factors, including robust screening programmes for infection in pregnancy, earlier HIV diagnosis and very high antiretroviral treatment (ART) coverage, with most pregnant people living with HIV already being on suppressive regimens at conception and earlier start of ART in pregnancy for those not already on treatment [4, 7]. Another key tool to reduce VT is administering postnatal prophylaxis (PNP) to the infant exposed to HIV. This was first demonstrated as effective in 1994 in the PACTG 076 clinical trial when zidovudine administered for 6 weeks to the newborn, along with a regimen consisting of zidovudine given antepartum and intrapartum to the mother, reduced the VT risk by approximately two‐thirds [8]. While PNP remains crucial for infants at high risk of VT, for those with a low risk, a reduced PNP regimen has been shown in several small studies to have a similar effectiveness to that of a longer regimen, with the added benefit of preventing unnecessary exposure of infants to antiretrovirals (ARVs) and potential associated toxicity [9, 10], and reducing medicalization of childbirth and infancy for families with low risk of VT. Since 2016, Swiss national guidelines have not recommended PNP for infants with the lowest risk of VT [11].

Infant feeding guidance is also essential in preventing VT. Although in most low‐ and middle‐income countries (LMICs) breastfeeding in the context of HIV is recommended to reduce infant mortality [12], in high‐income countries (HICs) with safe alternatives available, the recommendations have historically been to avoid breastfeeding, in order to eliminate the risk of postnatal VT [13, 14]. However, in the context of an undetectable viral load (VL) throughout breastfeeding, the VT rate has been demonstrated to be very low. The PROMISE clinical trial showed that, when the mother is on ART, the risk of VT was below 1% after 18 months of breastfeeding [15]. This, coupled with the desire of people in HICs living with HIV to breastfeed [15], has prompted debate among the scientific community and other key stakeholders as to whether the low VT risk when on effective ART during the breastfeeding period justifies excluding infants and birthing parents from the other potential lifelong benefits of breastfeeding [16, 17, 18]. Based on this, in recent years, there has been a policy shift, where the guidelines of many HICs have been modified to stress the importance of patient‐centred, evidence‐based counselling on infant feeding options, and to support families who wish to breastfeed as long as certain criteria are met and additional monitoring is in place [19, 20, 21, 22].

While the need to re‐evaluate PNP regimen choice has become apparent, many unanswered questions still surround the role of PNP and the optimum regimen for infants, partly due to the lack of clinical trial data [23]. In addition, clinical trial and cohort evidence in relation to PNP and infant feeding is predominantly from LMIC settings and is limited in European and other HIC settings with more extensive ART access and where more intensive birthing parent and infant monitoring is possible [23, 24]. Consequently, over time, policies and practice have diverged and now vary internationally [23, 25].

In this study, we conducted an online European survey of healthcare professionals with expertise in the prevention of VT, aiming to ascertain and summarize PNP and infant feeding policies and practices across Europe, in order to highlight any key differences and inform future harmonized guideline development.

## METHODS

An online survey was conducted between July and September 2023. Twenty countries were selected based on country size, burden of HIV, and obtaining a sufficient geographic spread of countries in the WHO European region: Belgium, Denmark, Estonia, France, Germany, Greece, Ireland, Israel, Italy, Latvia, the Netherlands, Poland, Portugal, Romania, the Russian Federation, Spain, Sweden, Switzerland, Ukraine and the UK. From the 20 chosen countries, 32 experts were invited to participate; they were identified through their membership of the Penta/European AIDS Clinical Society (EACS) HIV guidelines committee, membership of the Penta Child Health network or as a known paediatric infectious disease specialist with specific expertise in the field of HIV. Multiple experts were invited from within six countries (France, Germany, Italy, Spain, UK, Ukraine) to ensure we received at least one response from these key countries with larger numbers of infants born to people living with HIV, as well as to capture potential within‐country regional differences.

The survey underwent a pilot phase before its deployment to ensure validity, with subsequent adjustments and edits implemented as needed. The questionnaire consisted of 46 items, including specific questions on the following issues: whether a risk stratification approach is used to decide on the PNP regimen administered and details of this, recommendations for infant feeding, laboratory monitoring, and whether a surveillance system is in place for HIV in pregnancy (Appendix A1). Participants were also asked which local, regional or national guidelines are followed in their institution to prevent VT, with an option to upload an electronic copy of the guidelines, if available, to validate the survey responses.

An invitation letter with a personalized survey link was emailed directly to potential respondents using REDCap (Research Electronic Data Capture; Vanderbilt University) hosted at University College London [26, 27]. Respondents were able to save their responses and go back to edit or add information using their unique survey link. Participants who did not respond were sent a personalized reminder by email every 2 weeks. Following non‐response after two reminders, an alternative expert from that country was invited.

Descriptive analyses were conducted using Stata version 17 (Stata Corp, College Station, TX, USA). In some cases, survey respondents were contacted by email for clarifications. The results were then presented to the respondents and any inconsistencies were cross‐checked and amended as necessary.

### Ethics approval

Ethical approval was granted by University College London Research Ethics Committee (reference 3715/009). All survey respondents were required to give their consent to participate via REDCap prior to accessing the survey.

## RESULTS

**FIGURE 1:**
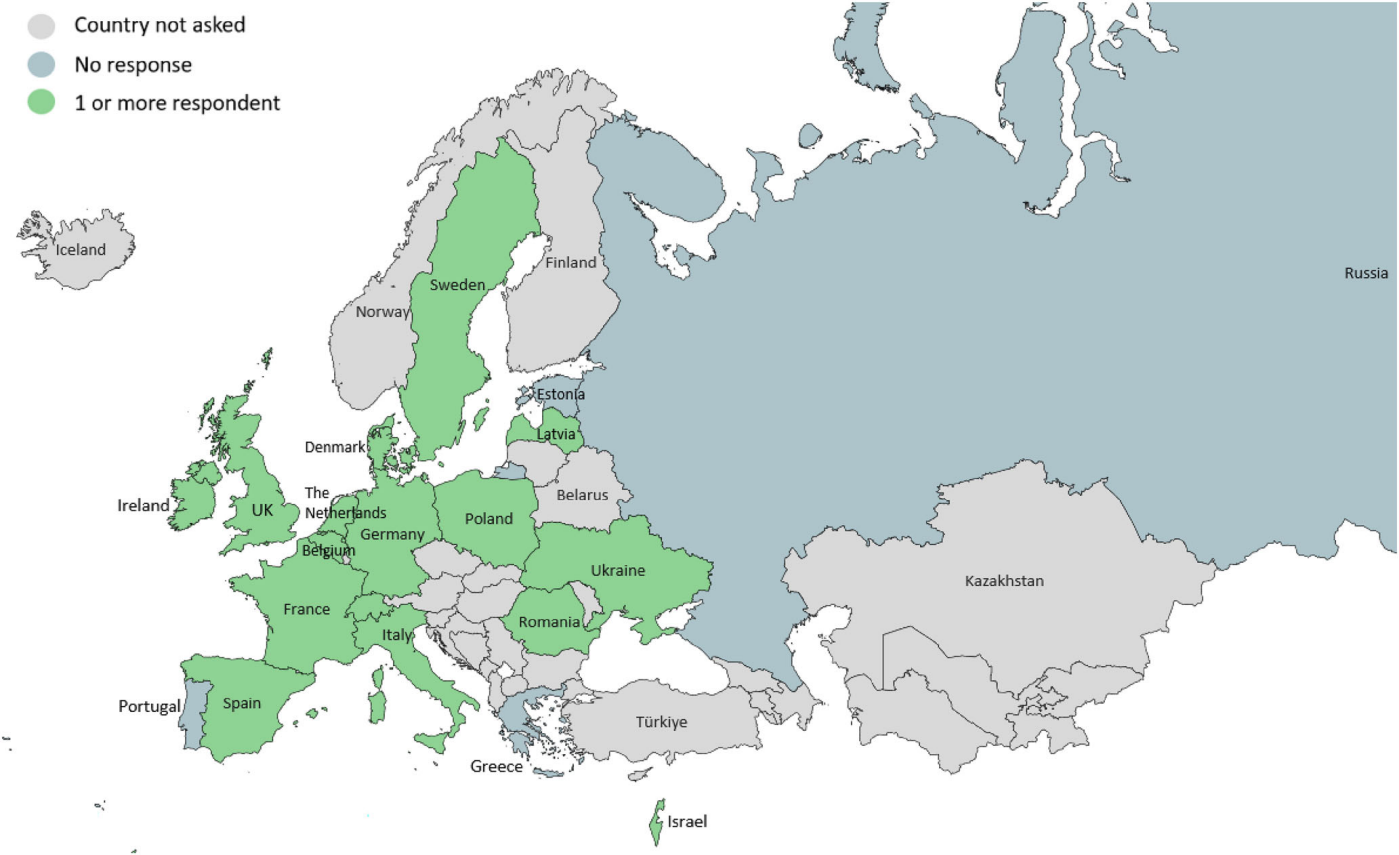
Participation among countries in the WHO European Region.

In all, 23 of the 32 invited experts responded, all of whom were paediatricians, representing 16/20 (80%) countries (Figure 1). Survey responses were not received from Estonia, Russia, Greece or Portugal. There were multiple respondents from the same country for Italy (*n* = 5), the UK (*n* = 2), Germany (*n* = 2) and France (*n* = 2). Sixteen respondents reported working in a centre where infants are born to people living with HIV. Of these, four reported an approximate annual number of one to 10 infants born in their centre, six reported 11–30, and three each reported 31–50 and 51–100. The remaining seven respondents indicated they were either directly involved in PNP decision‐making for individual cases, follow‐up testing of infants, and/or providing PNP guidance to other clinical providers.

National guidelines were reported to be available in all countries (15/16) apart from Latvia, which reported that local institutional guidelines were used in the respondent's institution, as well as guidelines from WHO, EACS and the British HIV Association. Guidelines were described as in the process of being updated in Ireland, France, the UK and Belgium.

### Postnatal prophylaxis

Risk stratification to guide choice of PNP regimen was reported for all countries. In nine out of 16 countries, three risk categories were reported, two risk categories were reported in six countries, and within‐country differences in categorization were reported for one country (Italy) (Table 1).

**TABLE 1:** Number of risk categories reported by survey respondents.

Two risk categories	Three risk categories
Switzerland	The Netherlands
France	Denmark
Sweden	Spain^b^
Ireland	UK
Belgium	Israel
Ukraine	Poland
Italy^a^	Latvia
	Romania
	Germany
	Italy^a^

^a^ Within‐country variation in practice reported in one out of four countries with multiple respondents.

^b^ The respondent indicated that, while three risk categories are followed locally, the national guidelines state two categories.

#### Lowest risk category

The criterion required to classify an infant into the lowest risk group common for all respondents was an undetectable VL during pregnancy; varying responses were described regarding the timing of this VL measurement, ranging from prior to and throughout pregnancy to ≥4 weeks prior to delivery. Additional criteria reported included: infant born full‐term, absence of breastfeeding, rupture of membranes <12 h, and no ART adherence concerns. For this lowest risk category, PNP duration ranged from no PNP to up to 4 weeks, with most countries recommending 2 weeks of PNP. The drug of choice reported was zidovudine, apart from one country (France), which reported nevirapine (Table 2).

**TABLE 2:** Postnatal prophylaxis (PNP) drug choice and duration used in participating countries for the lowest risk category.

Duration of PNP	No PNP	7–10 days	2 weeks	2–4 weeks	4 weeks
Respondent's country	Switzerland, Latvia, Italy^a^, Germany^a^	Spain^b^	UK, Germany^a^, Sweden, Israel, Poland, the Netherlands, Denmark, Italy^a^, France^c^	Italy^a^, Ukraine	Romania, Belgium, Ireland, Italy^a^

^a^ Within‐country variation in practice reported in two out of four countries with multiple respondents.

^b^ The respondent indicated that, while the local practice is to follow a duration of 7–10 days, the national guidelines recommend 2 weeks.

^c^ All responses indicated that zidovudine was used for the low‐risk category, apart from one which reported using nevirapine (France).

#### Middle risk category

Of the respondents who reported three PNP risk categories, the criteria used to classify an infant into the middle category included a maternal VL that was detectable but <1000 copies/mL, a VL <50 copies/mL in the second and third trimesters and at or after 36 weeks of gestation (but not in the first trimester), and a VL <50 copies/mL at or after 36 weeks of gestation only. Respondents from Romania and Italy (1/5) reported the use of two agents for this category: zidovudine plus lamivudine. All other responses indicated zidovudine alone. The duration of PNP varied from 3 days to 6 weeks.

#### Highest risk category

The criteria to classify an infant into the highest PNP risk category common across all respondents were no ART in pregnancy and/or detectable VL during pregnancy; varying responses were described regarding the timing of this VL measurement in pregnancy. Additional criteria included uncertainty regarding the birthing parent's ART adherence, complicated childbirth and acute HIV infection during pregnancy. The most common regimen reported for this category was zidovudine/lamivudine/nevirapine (20/23 respondents). Use of zidovudine/lamivudine/raltegravir was reported by two respondents (Israel and Italy, 1/5), whereas one country (Latvia) reported using two drugs as PNP for this category: zidovudine/nevirapine. The regimen duration varied from 2 to 6 weeks.

#### Drug dosing

Respondents from 10/16 countries (UK, 1/2 respondents; Germany, 1/2 respondents; Italy, 4/5 respondents) indicated that the doses of drugs recommended for PNP were the same as those recommended for the treatment of infants confirmed to have HIV infection (Data S1, Table S1). Of those who reported a different dose for prophylaxis compared with treatment, all who specified indicated a lower dose was used. ARVs reported to be modified were zidovudine, nevirapine and lamivudine.

#### Preterm

If the infant was born preterm, modifying the choice, dose or duration of PNP was recommended in most countries (*n* = 12). Belgium reported no modifications, and within‐country variation was reported in Italy and the UK. One country's response was missing for this question. Of the 18 responses that reported modifying PNP if the infant was born preterm, all indicated the dose of PNP was modified, five respondents reported modifying both dose and duration of PNP, and one reported modifying the dose and drug used for PNP.

### Infant feeding

Respondents in eight of the 16 countries indicated that the guidelines they follow support breastfeeding for infants born to people living with HIV; in the other eight, guidelines were reported as not supporting breastfeeding or not specifying (Table 3). The prerequisite criteria for supported breastfeeding included a VL of <50 copies/mL, with measurement timing when specified, varying from throughout pregnancy to two results 4 weeks apart in the third trimester. Additional criteria included good ART adherence, no plans to change the ARV regimen after childbirth or during breastfeeding, a willingness to undergo more intensive VL monitoring for both the breastfeeding person and infant, and good engagement with the multidisciplinary team. Recommendations for frequency of monitoring of the breastfeeding person varied from weekly at PNP cessation to, initially, monthly, and then every 2–3 months. Two of the 23 respondents reported a policy of extending the duration of PNP for breastfed infants: one from France indicated that nevirapine is prolonged for the entire breastfeeding period, while the other from Italy did not provide further details.

**TABLE 3:** Survey responses on breastfeeding by country.

Support breastfeeding	Do not support breastfeeding	Not specified
Switzerland	Belgium	Italy^a^
UK	Denmark	
Germany	Spain	
The Netherlands	France	
Poland	Israel	
Ukraine	Italy^a^	
Ireland	Latvia	
Sweden^b^	Romania	

^a^ Within‐country variation reported in one out of four countries with multiple respondents.

^b^ The original response to this question was ‘do not support breastfeeding’; however, the respondent later indicated that the recommendations had changed.

The eight countries with guidelines that support breastfeeding provided the following information on practices if a breastfeeding person is found to have a detectable VL. One respondent from Switzerland indicated that the guidelines would recommend stopping breastfeeding only. Respondents from Germany, Poland, Ukraine and the Netherlands (not formally included in their guidelines) would recommend stopping breastfeeding and conducting additional infant HIV testing. A respondent from Sweden would recommend stopping breastfeeding and considering post‐exposure prophylaxis (PEP). Respondents from the UK and Ireland would recommend stopping breastfeeding, performing additional infant testing and considering PEP.

### Infant testing

#### Non‐breastfed infants

**FIGURE 2:**
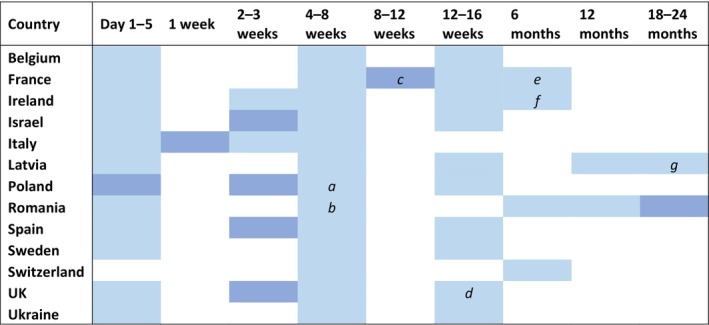
Participating countries HIV polymerase chain reaction (PCR) testing schedule for non‐breastfed infants (13/16 countries provided a response). Key: 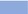 This test will be performed only for infants classified as being at high risk of vertical transmission. *a*, if the infant is classified as high risk for vertical transmission, the respondent indicated that this test would instead be conducted 2 weeks after stopping presumptive therapy; *b*, if the infant is classified as high‐risk, the respondent indicated this test would instead be conducted at 2 weeks; *c*, one out of two respondents indicated testing at this time point was optional; *d*, one out of two respondents reported this time point to be 10–12 weeks if low‐risk, and 12 weeks if high‐risk; *e*, this time point was reported by one out of two respondents only; *f*, the respondent indicated the test at this time point would not be performed if there were two prior negative PCRs; *g*, the respondent indicated that testing at this timepoint would only be conducted if necessary.

Of the 13 countries that provided a response, all reported using polymerase chain reaction (PCR) (Figure 2) and serology (Data S1, Figure S1) to diagnose or exclude HIV infection. The recommended total number of tests performed and timing of each test varied by country. Seven countries reported conducting additional PCR tests if the infant was identified as being at high risk of VT.

#### Breastfed infants

**FIGURE 3:**
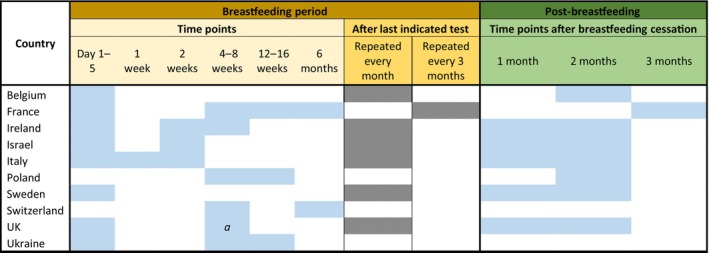
Participating countries HIV polymerase chain reaction testing schedule for breastfed infants (10/16 countries provided a response). *a*, one out of two respondents reported this time point to be 2 weeks.

The PCR testing schedule was modified if the infant was breastfed for all countries (10/16 countries provided a response for this question) except for two: Ukraine and Switzerland (Figure 3). The serology testing schedule for breastfed infants is shown in Data S1, Figure S2.

### National/regional surveillance systems

Five of the 16 countries were reported as not currently having national/regional surveillance systems for HIV in pregnancy (Italy, Poland, Latvia, Belgium and Switzerland).

## DISCUSSION

Although some general principles are consistent across all settings, the results of this survey demonstrate that guidelines and practice for PNP, infant feeding and monitoring vary substantially across Europe and also within some countries. This variation across Europe reflects the lack of recent clinical trial evidence relevant to the contemporary population of pregnant people living with HIV in Europe, where most are on fully suppressive ART [23, 24]. For example, most recommendations in the neonatal management section of the British HIV Association (BHIVA) pregnancy guidelines are based on low‐ or very low‐quality evidence, as assessed by the BHIVA modified Grading of Recommendations Assessment, Development and Evaluation (GRADE) system [20].

Vertical transmission rates in the countries surveyed are very low. For example, surveillance and cohort studies from England, Spain, France, Italy and Germany report a VT rate of 0.25%, 0.3%, 0.2%, 1.1% and 1.1%, respectively [1, 2, 3, 4, 5, 6]. However, slight differences in the VT rates as well as differences in the prevalence of HIV in pregnancy across European countries may contribute to the variation observed. Additional reasons for this variation may include differences in resources, such as access to frequent laboratory monitoring and drug formulations, and differences in how risks and benefits are considered in different countries [28, 29].

Several guidelines were in the process of being updated at the time of the survey. In the absence of clinical trial evidence, this evolution of recommendations is based on a number of considerations, including the extrapolation of evidence from LMICs as well as from studies on the sexual transmission of HIV [30], the increasing real‐world evidence on the very low risk of VT when ART is initiated prior to conception and continued throughout pregnancy with an undetectable VL, as well as expert opinion [23, 24, 31].

The survey results show that older agents such as zidovudine and nevirapine remain widely used for PNP across Europe, despite no longer being commonly used for HIV treatment as more robust, less toxic agents have been licensed. While ARVs for the treatment and prevention of HIV in adults have advanced, infant PNP has lagged behind [24]. Reasons for this include the difficulties in conducting clinical research in this population relating to age and associated unique ethical considerations [24], a lack of appropriate infant formulations [23], and the difficulties in ensuring safe and effective dosing of PNP as the infant rapidly grows in the first months of life [32]. However, as reported in the HPTN 040/PACTG 1043 trial, 8.4% of infants experienced serious adverse effects, mainly neutropenia and anaemia, possibly related to the PNP used. These haematological toxicities were significantly higher in the three‐drug group than the one‐ or two‐drug group [33]. Similarly, the European Pregnancy and Paediatric Infections Cohort Collaboration (EPPICC) reported that 6.7% and 9.1% of infants experienced grade 3–4 anaemia and neutropenia, respectively, in their first 6 months of life, and this was found to be associated with preterm delivery [34]. Due to these findings, there is a need to develop new PNP options that are both effective and have minimal toxicity and a high barrier to resistance, particularly for infants born preterm and those with a high risk of VT [35]. Potential new options in the pipeline include broadly neutralizing antibodies and long‐acting injectable agents. Clinical studies on these for use as PNP, including assessments of different durations of PNP, should be prioritized, as well as novel trial designs to study rare outcomes [24].

Several survey respondents reported no PNP for infants in the lowest risk group. In Switzerland, this policy change occurred in 2016. Justifications for this included that there is no randomized controlled trial evidence available to support PNP with zidovudine as a single prevention measure in the ‘optimal scenario’ (i.e. if a pregnant person had a very low VT risk), as well as based on the zero risk of sexual transmission with undetectable HIV VL [11], combined with concerns about toxicity and the desire to prevent the unnecessary exposure of infants to ARVs [23]. This policy has been shown to be widely adopted in Switzerland and has since been supported by the results of a Swiss study where 87 infants born between 2010 and 2018 to a person in the ‘optimal scenario’ were not prescribed PNP with no transmissions [36].

Rates of breastfeeding remain low but are increasing for people living with HIV in European countries in recent years [1, 14, 37, 38, 39]. Despite this, only half of the countries surveyed reported that their guidelines currently support breastfeeding. The recently published INSURE survey, which predominately focused on infant feeding recommendations across Europe, reported that the guidelines of 12 out of 25 of the countries surveyed between March and May 2022 recommend against breastfeeding [28]. Some differences between the results of this study and INSURE are apparent – for example, for Denmark (in the INSURE survey, reported as offering breastfeeding in certain cases), Ireland and Sweden (in the INSURE survey, reported as recommending against) and Israel (in the INSURE survey, reported as not applicable). Reasons for these differences may include: the timing of the surveys; evolution of breastfeeding policies; differences in the phrasing/interpretation of the survey questions; and differences in the clinician's interpretation of the guidelines. This Penta survey provides additional details, particularly on policies regarding PNP and infant testing.

Discouraging breastfeeding may contribute to inequities in health in this population, by increasing the prevalence of diabetes, postnatal depression, obesity and cancer among the birthing parent, and increasing the risk of asthma, obesity, severe lower respiratory tract infections, atopic dermatitis and diabetes in their infants [33]. Recently updated EACS guidelines [40] support breastfeeding, provided specific criteria are met (optimal maternal ART adherence, fully suppressed VL, availability of regular multidisciplinary team support, and VL monitoring); this approach may further increase supported breastfeeding for those living with HIV in Europe. However, further research should be prioritized as there are many unanswered questions on the mechanisms and risk factors for breast milk transmission. For example, in the pre‐ART era, mastitis, infant gastroenteritis, infant thrush and mixed feeding were identified as risk factors, and it is unclear if this is still the case for those on suppressive ART [41, 42, 43]. Moreover, as most of the studies have come from LMICs, it is uncertain whether these can be generalized to HICs [44, 45]. For example, data extrapolated from LMICs may overestimate the risk of VT in HICs due to differences between the two in the prevalence of contributory factors such as infant gastrointestinal infection [25, 46].

Two survey respondents indicated extending PNP if the infant is breastfed. The PROMISE trial reported that PNP given daily to the infant and ART given to the breastfeeding person were equally effective in reducing VT during breastfeeding [15]. Questions remain about the added benefit of PNP for a breastfed infant when the breastfeeding person is receiving effective ART [46, 47].

It is essential that changes in PNP use and infant feeding practices, alongside transmission data, are monitored through national surveillance systems such as the Integrated Screening Outcomes Surveillance Service in England [1]. Any transmissions should be identified, and the circumstances under which they occur should be investigated. To compare outcomes of different practices across Europe and to inform future best practices, findings from EPPICC, which pools data from multiple cohorts across Europe, will be crucial. This is because such studies provide larger sample sizes and greater statistical power [48]. Ultimately, based on emerging evidence, European countries should aim to reach general consensus on guidance regarding PNP and infant feeding through harmonizing guidelines, although it is recognized that this may not always be appropriate due to differences in the populations of pregnant people living with HIV across Europe as well as the availability of resources. Although evidence demonstrates that the risk of VT during breastfeeding in the context of ART and viral suppression is very low, there is currently insufficient evidence to state that the risk is zero [18, 46, 49]. It is essential that families and people planning pregnancies are kept informed of the current evidence and that principles of shared decision‐making are followed when considering choices around infant feeding.

As ongoing improvements in testing, ART and the care of pregnant people with HIV continue to reduce VT rates, it is vital that guidelines acknowledge the preferences of people living with HIV. For example, the Nourish‐UK study involves pregnant people living with HIV in the discussions regarding infant feeding; these personal narratives will then be used to inform new policy and guidance in the UK [50, 51]. Furthermore, care should be taken to minimize unnecessary medicalization of pregnancy, childbirth and infant feeding and to reduce infant drug exposure as much as possible without increasing the VT risk.

### Limitations

As this was a cross‐sectional survey undertaken in July–September 2023, it does not take into account the fact that country guidelines are updated at different times, and thus some of the described instances of guideline variation across countries may partly reflect the timing of such updates. The survey results were reviewed by only two members of the study team, which could introduce bias. Including an independent reviewer with relevant expertise, external to both the survey respondents and the study team, could have mitigated this potential bias. Most respondents were identified based on their membership of the Penta/EACS HIV guidelines committee or the Penta network. Therefore, their country guidelines may have been more similar to each other than to the countries not invited to participate which have a lower prevalence of HIV in pregnancy and consequently may have less local expertise available. Survey responses were not received for four countries.

## CONCLUSIONS

There are important differences across Europe in relation to PNP and infant feeding policies and practices. Due to the lack of clinical trial evidence, data from large‐scale pooled observational studies will be crucial to inform future practice. Countries without national surveillance for HIV in pregnancy and their infants should consider establishing such systems and collaborating with existing cohorts. The differences and similarities across countries described in this survey can help to contribute to the interpretation of such data. Effort is needed across Europe to align policies and practice to reflect the most up‐to‐date knowledge to ensure the risk of VT is minimized and unnecessary PNP avoided, while at the same time supporting families to make informed decisions on infant feeding and normalizing the processes of pregnancy, childbirth and infant feeding.

## AUTHOR CONTRIBUTIONS

GF, AB, CT, EC, CRK and TG designed the questionnaire. GF and AB analysed the survey results. GF, AB, CT and EC contributed to writing of the manuscript. CRK, TG, SA, SB, GC, EC, CD, PF, BF, LG, VG, GGS, CK, HL, MM, MM, LN, TN, ANJ, KS, AV and SW contributed to the survey and validated the results. All authors reviewed and approved the final version.

## FUNDING INFORMATION

GF holds a PhD studentship funded by the Fondazione Penta ETS. This work is supported by the NIHR GOSH BRC. The views expressed are those of the author(s) and not necessarily those of the NHS, the NIHR or the Department of Health.

## CONFLICT OF INTEREST STATEMENT

CT received funding to the Penta Foundation from ViiV Healthcare and MSD. CD received funding from ViiV to attend the IAS 2023 conference and funding from MSD for an oral presentation at a symposium 2023. PF received grants from ANRS‐MIE which were paid to his institution, personal fees from MSD France, ViiV Healthcare, Janssen Cilag, Gilead Sciences, support for attending meetings/and or travel from MSD France, Gilead Sciences and ViiV Healthcare. All other authors declare they have no competing interests.

## Supporting information


**Data S1.** Supporting information.

**Appendix A1.** European survey of policies for postnatal prophylaxis and infant feeding for prevention of vertical HIV transmission.

## Data Availability

The data that support the findings of this study are available from the corresponding author upon reasonable request.
