# Supplementary material for: HIV postnatal prophylaxis and infant feeding policies vary across Europe: results of a Penta survey
Source: HIV Med. 2024 Oct 23;26(2):207–17. doi: 10.1111/hiv.13723 (PMC11786621; doi:10.1111/hiv.13723)
Supplement: Supplementary file 1 — Data S1. Supporting information. [file HIV-26-207-s001.docx]

**Supplementary material**

**Table S1. Countries with guidelines recommending the same doses of drugs for PNP as used for the treatment of HIV in infants**

| **Same drug doses used for PNP as used for treatment** | **Different drug doses used for PNP compared to those for treatment** | **Not specified** |
| --- | --- | --- |
| Switzerland | Sweden | Denmark |
| UK† | UK†‡ | Latvia |
| Romania | Spain |  |
| Ireland | France |  |
| Poland | Ukraine |  |
| Italy† | Italy† |  |
| Israel | The Netherlands§ |  |
| Belgium | Germany† |  |
| Germany† |  |  |

† Within country variation reported in 3/4 countries with multiple respondents.

‡ One respondent indicated in the next iteration of guidelines treatment doses will be recommended.

§ The respondent indicated that the Dutch guidelines do not include dosing recommendations, and instead, the Dutch Paediatric Formulary (kinderformularium) is used for dosing guidance.

| **Country** | **Day 1-3** | **2 weeks** | **6 weeks** | **4 months** | **6 months** | **12-18 months** | **18-24 months** |
| --- | --- | --- | --- | --- | --- | --- | --- |
| **Belgium** |  |  |  |  |  |  |  |
| **France** |  |  |  |  |  |  |  |
| **Ireland** |  |  |  |  |  |  |  |
| **Israel** |  |  |  |  |  |  |  |
| **Italy** |  |  |  |  |  |  |  |
| **Latvia** |  |  |  |  |  |  | *c* |
| **Poland** |  |  |  |  |  |  |  |
| **Romania** |  |  |  |  |  |  |  |
| **Spain** |  |  |  |  |  |  |  |
| **Sweden** |  |  |  |  |  |  |  |
| **Switzerland** |  |  |  |  |  |  |  |
| **UK** | *a* |  |  |  |  |  |  |
| **Ukraine** | *b* |  |  |  |  |  | *d* |

**Figure S1. Participating countries HIV serology testing schedule for non-breastfed infants (13/16 countries provided a response).**

**Key**

This test will be performed only for infants classified as being at high risk of vertical transmission.

**
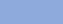
**

|  |  |
| --- | --- |

*a* - Testing at this time point was reported by 1/2 respondents who said it would only be performed if the birthing parent’s antibody status was not documented.

*b* - The respondent indicated testing at this time point would only be performed if the birthing parent’s antibody status was not documented.

*c* - The respondent indicated that testing at this timepoint would only be conducted if necessary.

*d* - The respondent indicated that a test will be performed at 18 months and repeated at 22-24 months if the previous result was positive.

| **Country** | **Day 1-3** |  | **18-24 months** |
| --- | --- | --- | --- |
| **Belgium** |  |  | *c* |
| **France** |  |  |  |
| **Ireland** |  |  |  |
| **Israel** |  |  | *c* |
| **Italy** |  |  | *c* |
| **Poland** |  |  |  |
| **Sweden** |  |  | *c* |
| **Switzerland** |  |  | *d* |
| **UK** | *a* |  | *c* |
| **Ukraine** | *b* |  | *e* |

**Figure S2. Participating countries HIV serology testing schedule for breastfed infants (10/16 countries provided a response).**

| *a* - Testing at this time point was reported by 1/2 respondents who indicated it would only be performed if the birthing parent’s antibody status was not documented. |
| --- |
| *b* - The respondent indicated testing at this time point would only be performed if the birthing parent’s antibody status was not documented. |
| *c* - Or a minimum of 8 weeks after breastfeeding cessation if this is later. |
| *d* - Or a minimum of 6 weeks after breastfeeding cessation if this is later. |
| *e* - Or a minimum of 12 weeks after breastfeeding cessation if this is later. |
