# Supplementary material for: HIV postnatal prophylaxis and infant feeding policies vary across Europe: results of a Penta survey
Source: HIV Med. 2024 Oct 23;26(2):207–17. doi: 10.1111/hiv.13723 (PMC11786621; doi:10.1111/hiv.13723)
Supplement: Supplementary file 2 — Appendix A1. European survey of policies for postnatal prophylaxis and infant feeding for prevention of vertical HIV transmission. [file HIV-26-207-s002.pdf]

European survey of policies for postnatal prophylaxis and infant feeding for prevention of vertical HIV transmission

Thank you for considering taking part in this survey. A participant information sheet was attached to the email where you accessed this survey link. To participate in the survey, please consent to the following statements. You will then be able to access the survey itself.

I confirm that I have read and understood the Information Sheet provided, and consent to participate in the survey.	◯ I confirm
I understand that my personal information (name, email address) may be used to contact me to ask for clarifications or with respect to potential publications, and that according to data protection legislation, 'public task' will be the lawful basis for processing.	◯ I confirm
I understand that my responses will be kept strictly confidential, and it will not be possible to identify me in any publications. However, if I indicate within the survey that I would like to be involved in potential publications, and my name is included in a report as a co-author, it may be possible to link my name to my countries policy information.	◯ I confirm
I understand that my participation is voluntary and that I am free to withdraw up to 2 weeks after the survey has closed without giving a reason.	◯ I confirm
I understand that the data will not be made available to any commercial organisations but is solely the responsibility of the researcher(s) undertaking this study.	◯ I confirm
I understand that the information I have submitted will be published as an abstract and/or paper and I will be given the option of being involved in potential publications.	◯ I confirm
I understand that my data gathered in this study will be stored securely and destroyed after 5 years.	◯ I confirm
Full name of the person completing the survey	__________________________________

Your email address	__________________________________
What is your job title?	◯ Paediatric infectious diseases doctor<br>◯ Paediatric doctor<br>◯ Infectious diseases doctor<br>◯ Obstetrics and gynaecology doctor<br>◯ Adult HIV doctor<br>◯ Virologist<br>◯ Other
Please specify "Other".	__________________________________

In which country do you work?

- ◯ Aaland Islands (AX)
- ◯ Afghanistan (AF)
- ◯ Albania (AL)
- ◯ Algeria (DZ)
- ◯ American Samoa (AS)
- ◯ Andorra (AD)
- ◯ Angola (AO)
- ◯ Anguilla (AI)
- ◯ Antarctica (AQ)
- ◯ Antigua and Barbuda (AG)
- ◯ Argentina (AR)
- ◯ Armenia (AM)
- ◯ Aruba (AW)
- ◯ Australia (AU)
- ◯ Austria (AT)
- ◯ Azerbaijan (AZ)
- ◯ Bahamas (BS)
- ◯ Bahrain (BH)
- ◯ Bangladesh (BD)
- ◯ Barbados (BB)
- ◯ Belarus (BY)
- ◯ Belgium (BE)
- ◯ Belize (BZ)
- ◯ Benin (BJ)
- ◯ Bermuda (BM)
- ◯ Bhutan (BT)
- ◯ Bolivia (BO)
- ◯ Bosnia and Herzegovina (BA)
- ◯ Botswana (BW)
- ◯ Bouvet Island (BV)
- ◯ Brazil (BR)
- ◯ British Indian Ocean Territory (IO)
- ◯ British Virgin Islands (VG)
- ◯ Brunei Darussalam (BN)
- ◯ Bulgaria (BG)
- ◯ Burkina Faso (BF)
- ◯ Burundi (BI)
- ◯ Cambodia (KH)
- ◯ Cameroon (CM)
- ◯ Canada (CA)
- ◯ Cape Verde (CV)
- ◯ Cayman Islands (KY)
- ◯ Central African Republic (CF)
- ◯ Chad (TD)
- ◯ Chile (CL)
- ◯ China (CN)
- ◯ Christmas Island (CX)
- ◯ Cocos (Keeling) Islands (CC)
- ◯ Colombia (CO)
- ◯ Comoros (KM)
- ◯ Congo Brazzaville (CG)
- ◯ Congo Kinshasa (CD)
- ◯ Cook Islands (CK)
- ◯ Costa Rica (CR)
- ◯ Cote D'Ivoire (CI)
- ◯ Croatia (HR)
- ◯ Cuba (CU)
- ◯ Curacao (CW)
- ◯ Cyprus (CY)
- ◯ Czech Republic (CZ)
- ◯ Denmark (DK)
- ◯ Djibouti (DJ)
- ◯ Dominica (DM)
- ◯ Dominican Republic (DO)
- ◯ Dronning Maud Land (NQ)
- ◯ Ecuador (EC)
- ◯ Egypt (EG)
- ◯ El Salvador (SV)
- ◯ Equatorial Guinea (GQ)

◯ Eritrea (ER)
◯ Estonia (EE)
◯ Ethiopia (ET)
◯ Falkland Islands (Malvinas) (FK)
◯ Faroe Islands (FO)
◯ Fiji (FJ)
◯ Finland (FI)
◯ France (FR)
◯ French Guiana (GF)
◯ French Polynesia (PF)
◯ French Southern Territories (TF)
◯ Gabon (GA)
◯ Gambia (GM)
◯ Georgia (GE)
◯ Germany (DE)
◯ Ghana (GH)
◯ Gibraltar (GI)
◯ Greece (GR)
◯ Greenland (GL)
◯ Grenada (GD)
◯ Guadeloupe (GP)
◯ Guam (GU)
◯ Guatemala (GT)
◯ Guernsey (GG)
◯ Guinea (GN)
◯ Guinea-Bissau (GW)
◯ Guyana (GY)
◯ Haiti (HT)
◯ Heard And McDonald Islands (HM)
◯ Honduras (HN)
◯ Hong Kong (HK)
◯ Hungary (HU)
◯ Iceland (IS)
◯ India (IN)
◯ Indonesia (ID)
◯ Iran (IR)
◯ Iraq (IQ)
◯ Ireland (IE)
◯ Isle of Man (IM)
◯ Israel (IL)
◯ Italy (IT)
◯ Jamaica (JM)
◯ Japan (JP)
◯ Jersey (JE)
◯ Jordan (JO)
◯ Kazakhstan (KZ)
◯ Kenya (KE)
◯ Kiribati (KI)
◯ Korea North (KP)
◯ Korea South (KR)
◯ Kuwait (KW)
◯ Kyrgyzstan (KG)
◯ Laos (LA)
◯ Latvia (LV)
◯ Lebanon (LB)
◯ Lesotho (LS)
◯ Liberia (LR)
◯ Libya (LY)
◯ Liechtenstein (LI)
◯ Lithuania (LT)
◯ Luxembourg (LU)
◯ Macau (MO)
◯ Macedonia (MK)
◯ Madagascar (MG)
◯ Malawi (MW)
◯ Malaysia (MY)
◯ Maldives (MV)
◯ Mali (ML)
◯ Malta (MT)
◯ Marshall Islands (MH)
◯ Martinique (MQ)

◯ Mauritania (MR)
◯ Mauritius (MU)
◯ Mayotte (YT)
◯ Mexico (MX)
◯ Micronesia (FM)
◯ Moldova (MD)
◯ Monaco (MC)
◯ Mongolia (MN)
◯ Montenegro (ME)
◯ Montserrat (MS)
◯ Morocco (MA)
◯ Mozambique (MZ)
◯ Myanmar (MM)
◯ Namibia (NA)
◯ Nauru (NR)
◯ Nepal (NP)
◯ Netherlands (NL)
◯ Netherlands Antilles (AN)
◯ New Caledonia (NC)
◯ New Zealand (NZ)
◯ Nicaragua (NI)
◯ Niger (NE)
◯ Nigeria (NG)
◯ Niue (NU)
◯ Norfolk Island (NF)
◯ Northern Mariana Islands (MP)
◯ Norway (NO)
◯ Oman (OM)
◯ Pakistan (PK)
◯ Palau (PW)
◯ Palestinian Territory (PS)
◯ Panama (PA)
◯ Papua New Guinea (PG)
◯ Paraguay (PY)
◯ Peru (PE)
◯ Philippines (PH)
◯ Pitcairn Islands (PN)
◯ Poland (PL)
◯ Portugal (PT)
◯ Puerto Rico (PR)
◯ Qatar (QA)
◯ Reunion (RE)
◯ Romania (RO)
◯ Russia (RU)
◯ Rwanda (RW)
◯ Saint Barthelemy (BL)
◯ Saint Helena, Ascension and Tristan da Cunha (SH)
◯ Saint Kitts and Nevis (KN)
◯ Saint Lucia (LC)
◯ Saint Martin (MF)
◯ Saint Pierre and Miquelon (PM)
◯ Saint Vincent and the Grenadines (VC)
◯ Samoa (WS)
◯ San Marino (SM)
◯ Sao Tome and Principe (ST)
◯ Saudi Arabia (SA)
◯ Senegal (SN)
◯ Serbia (CS)
◯ Seychelles (SC)
◯ Sierra Leone (SL)
◯ Singapore (SG)
◯ Slovakia (SK)
◯ Slovenia (SI)
◯ Solomon Islands (SB)
◯ Somalia (SO)
◯ South Africa (ZA)
◯ South Georgia And The South Sandwich Islands (GS)
◯ Spain (ES)
◯ Sri Lanka (LK)
◯ Sudan (SD)
◯ Suriname (SR)

◯ Svalbard (SJ)
◯ Swaziland (SZ)
◯ Sweden (SE)
◯ Switzerland (CH)
◯ Syria (SY)
◯ Taiwan (TW)
◯ Tajikistan (TJ)
◯ Tanzania (TZ)
◯ Thailand (TH)
◯ Timor-Leste (East Timor) (TL)
◯ Togo (TG)
◯ Tokelau (TK)
◯ Tonga (TO)
◯ Trinidad and Tobago (TT)
◯ Tunisia (TN)
◯ Turkey (TR)
◯ Turkmenistan (TM)
◯ Turks and Caicos Islands (TC)
◯ Tuvalu (TV)
◯ U.S. Virgin Islands (VI)
◯ Uganda (UG)
◯ Ukraine (UA)
◯ United Arab Emirates (AE)
◯ United Kingdom (GB)
◯ United States (US)
◯ United States Minor Outlying Islands (UM)
◯ Uruguay (UY)
◯ Uzbekistan (UZ)
◯ Vanuatu (VU)
◯ Vatican City (VA)
◯ Venezuela (VE)
◯ Vietnam (VN)
◯ Wallis And Futuna Islands (WF)
◯ Western Sahara (EH)
◯ Yemen (YE)
◯ Zambia (ZM)
◯ Zimbabwe (ZW)
(you can type the country in)

What is the name of the hospital(s) where you work?

____

What is the name of the city/town where this hospital(s) is located?

____

In your country, which of the following do you have available to guide practice for postnatal prophylaxis?

Select all that apply

☐ National guidelines
☐ Regional guidelines
☐ Local institutional guidelines
☐ Guidelines from another country or organisation (e.g. WHO)
☐ No guidelines
☐ Other

Please specify "Guidelines from another country or organisation (e.g. WHO)".

____

Please specify "Other".

____

Which of these guidelines are used in your centre?<br><br>Select all that apply	☐ National guidelines<br>☐ Regional guidelines<br>☐ Local institutional guidelines<br>☐ Guidelines from another country or organisation (e.g. WHO)<br>☐ No guidelines<br>☐ Other
If you have national guidelines but also use regional or local guidelines, are there any major differences between them?	◯ Yes<br>◯ No<br>◯ Not applicable
Please outline the main differences.	__________________________________
Please either provide the reference (PubMed ID or Digital Object Identifier (DOI) or URL) of the guidelines/recommendations you follow...	__________________________________
...or upload the documents<br><br>Doc 1 ______<br>Doc 2 ______<br>Doc 3 ______<br>Doc 4 ______<br>Doc 5 ______<br>Doc 6 ______	
Do you work in a centre where infants are born to women living with HIV?	◯ Yes<br>◯ No
Approximately how many infants at risk of vertical HIV transmission (i.e. born to a mother with HIV) are born in your centre per year.	◯ 1-10<br>◯ 11-30<br>◯ 31-50<br>◯ 51-100<br>◯ >100<br>◯ I don't know
Are you directly involved in decision-making on postnatal prophylaxis recommendations for individual cases?	◯ Yes<br>◯ No
Are you directly involved in the follow-up testing of infants at risk of vertical transmission of HIV (i.e. born to a mother with HIV)?	◯ Yes<br>◯ No
Are you involved in providing general guidance to other clinical providers on postnatal prophylaxis?	◯ Yes<br>◯ No
At which level is this?<br><br>Select all that apply	☐ Institutional<br>☐ Regional<br>☐ National<br>☐ International<br>☐ Other
Please specify "Other".	__________________________________

Do you use a risk stratification approach to decide which postnatal prophylaxis, if any, to give to infants born to mothers living with HIV?	◯ Yes<br>◯ No
Which drug/s are routinely recommended for postnatal prophylaxis?<br><br>Select all that apply	☐ Zidovudine<br>☐ Lamivudine<br>☐ Nevirapine<br>☐ Abacavir<br>☐ Tenofovir-DF<br>☐ Lopinavir/ritonavir<br>☐ Raltegravir<br>☐ Other
Please specify "Other".	__________________________________
For how many weeks is this drug/s given? If different drugs are given for different durations, please explain.	____
How many risk categories are there?	◯ 2<br>◯ 3<br>◯ 4
What is the title of category 1 (e.g. high risk, low risk or very low risk)?	__________________________________
Please state the criteria used to classify an infant into this category.	____
How many (if any) different postnatal prophylaxis drugs are given to an infant in category 1?	◯ No postnatal prophylaxis drugs used<br>◯ Single drug postnatal prophylaxis used<br>◯ Combination postnatal prophylaxis drugs used
When a single drug is used for postnatal prophylaxis, which one do you most routinely recommend?	◯ Zidovudine<br>◯ Lamivudine<br>◯ Nevirapine<br>◯ Abacavir<br>◯ Tenofovir-DF<br>◯ Lopinavir/ritonavir<br>◯ Raltegravir<br>◯ Other
Please specify "Other".	__________________________________
For how many weeks is this drug given?	__________________________________
When a combination of postnatal prophylaxis drugs are used, which ones do you most routinely recommend?<br><br>Select all that apply	☐ Zidovudine<br>☐ Lamivudine<br>☐ Nevirapine<br>☐ Abacavir<br>☐ Tenofovir-DF<br>☐ Lopinavir/ritonavir<br>☐ Raltegravir<br>☐ Other

Please specify "Other".	__________________________________
For how many weeks are these drugs given? If different drugs are given for different durations, please explain.	____
What is the title of category 2 (e.g. high risk, low risk or very low risk)?	__________________________________
Please state the criteria used to classify an infant into this category.	____
How many (if any) different postnatal prophylaxis drugs are given to an infant in category 2?	◯ No postnatal prophylaxis drugs used<br>◯ Single drug postnatal prophylaxis used<br>◯ Combination postnatal prophylaxis drugs used
When a single drug is used for postnatal prophylaxis, which one do you most routinely recommend?	◯ Zidovudine<br>◯ Lamivudine<br>◯ Nevirapine<br>◯ Abacavir<br>◯ Tenofovir-DF<br>◯ Lopinavir/ritonavir<br>◯ Raltegravir<br>◯ Other
Please specify "Other".	__________________________________
For how many weeks is this drug given?	__________________________________
When a combination of postnatal prophylaxis drugs are used, which ones do you most routinely recommend?<br><br>Select all that apply	☐ Zidovudine<br>☐ Lamivudine<br>☐ Nevirapine<br>☐ Abacavir<br>☐ Tenofovir-DF<br>☐ Lopinavir/ritonavir<br>☐ Raltegravir<br>☐ Other
Please specify "Other".	__________________________________
For how many weeks are these drugs given? If different drugs are given for different durations, please explain.	____
What is the title of category 3 (e.g. high risk, low risk or very low risk)?	__________________________________
Please state the criteria used to classify an infant into this category.	____

How many (if any) different postnatal prophylaxis drugs are given to an infant in category 3?	◯ No postnatal prophylaxis drugs used<br>◯ Single drug postnatal prophylaxis used<br>◯ Combination postnatal prophylaxis drugs used
When a single drug is used for postnatal prophylaxis, which one do you most routinely recommend?	◯ Zidovudine<br>◯ Lamivudine<br>◯ Nevirapine<br>◯ Abacavir<br>◯ Tenofovir-DF<br>◯ Lopinavir/ritonavir<br>◯ Raltegravir<br>◯ Other
Please specify "Other".	__________________________________
For how many weeks is this drug given?	__________________________________
When a combination of postnatal prophylaxis drugs are used, which ones do you most routinely recommend?<br><br>Select all that apply	☐ Zidovudine<br>☐ Lamivudine<br>☐ Nevirapine<br>☐ Abacavir<br>☐ Tenofovir-DF<br>☐ Lopinavir/ritonavir<br>☐ Raltegravir<br>☐ Other
Please specify "Other".	__________________________________
For how many weeks are these drugs given? If different drugs are given for different durations, please explain.	____
What is the title of category 4 (e.g. high risk, low risk or very low risk)?	__________________________________
Please state the criteria used to classify an infant into this category.	____
How many (if any) different postnatal prophylaxis drugs are given to an infant in category 4?	◯ No postnatal prophylaxis drugs used<br>◯ Single drug postnatal prophylaxis used<br>◯ Combination postnatal prophylaxis drugs used
When a single drug is used for postnatal prophylaxis, which one do you most routinely recommend?	◯ Zidovudine<br>◯ Lamivudine<br>◯ Nevirapine<br>◯ Abacavir<br>◯ Tenofovir-DF<br>◯ Lopinavir/ritonavir<br>◯ Raltegravir<br>◯ Other
Please specify "Other".	__________________________________

For how many weeks is this drug given?

____

When a combination of postnatal prophylaxis drugs are used, which ones do you most routinely recommend?

Select all that apply

☐ Zidovudine
☐ Lamivudine
☐ Nevirapine
☐ Abacavir
☐ Tenofovir-DF
☐ Lopinavir/ritonavir
☐ Raltegravir
☐ Other

Please specify "Other".

____

For how many weeks are these drugs given? If different drugs are given for different durations, please explain.

____

Are the doses of drugs recommended for postnatal prophylaxis the same as those recommended for treatment of confirmed HIV in infants?

◯ Yes
◯ No

Please specify how the drug dosing differs.

____

Is a modification to choice, dose or duration of postnatal prophylaxis recommended in the case of prematurity?

◯ Yes
◯ No

Please summarise the modification.

____

Do the guidelines you follow allow/support the breastfeeding of an infant born to a mother living with HIV?

◯ Yes
◯ No
◯ Not specified

Please state the prerequisite maternal criteria in which breastfeeding with HIV is allowed/supported.

____

If a decision to breastfeed is made, what are the recommendations in relation to the laboratory monitoring of the mother during breastfeeding?

____

Are the recommendations for postnatal prophylaxis different for a breast-fed infant compared to a non-breast-fed infant?

◯ Yes
◯ No

Please specify the differences.

____

At which time points is a non-breast-fed infant tested for HIV, and which tests are used at each time point (e.g. RNA PCR, DNA PCR, Ab/Ag)?	__________________________________
At which time points is a breast-fed infant tested for HIV, and which tests are used at each time point (e.g. RNA PCR, DNA PCR, Ab/Ag)?	__________________________________
If a breastfeeding mother is found to have a detectable viral load, what are the recommendations in terms of infant feeding, testing and postnatal prophylaxis?	__________________________________
If not included in the guidelines previously uploaded, please upload the guidelines/recommendations you follow in relation to the breastfeeding of an infant born to a mother with HIV.	
Is there a national and/or regional surveillance system for HIV in pregnancy in your country?	◯ Yes<br>◯ No
Please provide a link to the website or provide the contact details of the national and/or regional surveillance system.	__________________________________
Would you like to be involved in potential publications for this survey?	◯ Yes<br>◯ No
Any comments?	__________________________________
